# Crystal structure, Hirshfeld surface analysis and computational study of bis­(2-{[(2,6-di­chloro­benzyl­idene)hydrazinyl­idene]meth­yl}phenolato)cobalt(II) and of the copper(II) analogue

**DOI:** 10.1107/S2056989019016529

**Published:** 2020-01-01

**Authors:** Rohit B. Manawar, Mayank J. Mamtora, Manish K. Shah, Mukesh M. Jotani, Edward R. T. Tiekink

**Affiliations:** a Chemical Research Laboratory, Department of Chemistry, Saurashtra University, Rajkot, Gujarat 360005, India; b Department of Physics, Bhavan’s Sheth R. A. College of Science, Ahmedabad, Gujarat 380001, India; c Research Centre for Crystalline Materials, School of Science and Technology, Sunway University, 47500 Bandar Sunway, Selangor Darul Ehsan, Malaysia

**Keywords:** crystal structure, Schiff base complex, cobalt, copper, Hirshfeld surface analysis, computational chemistry

## Abstract

The title homoleptic Schiff base complexes, [*M*(C_14_H_9_Cl_2_N_2_O)_2_], for *M* = Co^II^, (I), and Cu^II^, (II), present distinct coordination geometries despite the Schiff base dianion coordinating *via* the phenolato-O and imine-N atoms in each case. For (I), the coordination geometry is based on a trigonal bipyramid whereas for (II), a square-planar geometry is found (Cu site symmetry ). In the crystal of (I), discernible supra­molecular layers in the *ac* plane are sustained by chloro­benzene-C—H⋯O(coordinated), chloro­benzene-C—H⋯π(fused-benzene ring) as well as π(fused-benzene, chloro­benzene)–π(chloro­benzene) inter­actions [inter-centroid separations = 3.6460 (17) and 3.6580 (16) Å, respectively]. The layers inter-digitate along the *b*-axis direction and are linked by di­chloro­benzene-C—H⋯π(fused-benzene ring) and π–π inter­actions between fused-benzene rings and between chloro­benzene rings [inter-centroid separations = 3.6916 (16) and 3.7968 (19) Å, respectively] . Flat, supra­molecular layers are also found in the crystal of (II), being stabilized by π–π inter­actions formed between fused-benzene rings and between chloro­benzene rings [inter-centroid separations = 3.8889 (15) and 3.8889 (15) Å, respectively]; these stack parallel to [10] without directional inter­actions between them. The analysis of the respective calculated Hirshfeld surfaces indicate diminished roles for H⋯H contacts [26.2% (I) and 30.5% (II)] owing to significant contributions by Cl⋯H/H⋯Cl contacts [25.8% (I) and 24.9% (II)]. Minor contributions by Cl⋯Cl [2.2%] and Cu⋯Cl [1.9%] contacts are indicated in the crystals of (I) and (II), respectively. The inter­action energies largely arise from dispersion terms; the aforementioned Cu⋯Cl contact in (II) gives rise to the most stabilizing inter­action in the crystal of (II).

## Abstract 2

Distinct coordination geometries are found in the crystals of the title Co^II^ (trigonal bipyramidal) and Cu^II^ (square-planar) complexes, each defined by a N_2_S_2_ donor set derived from two chelating Schiff base anions.

## Chemical context   

Schiff base mol­ecules are well-known ligands because of the ease of their formation and for their rich coordination chemistry with a wide range of metal ions. A prominent application of metal–Schiff base complexes is as catalysts in different chemical reactions (Patti *et al.*, 2009 ▸). The Schiff base mol­ecules themselves are of considerable inter­est as they display a broad range of biological activities such as anti-bacterial, anti-fungal, anti-viral, anti-malarial, anti-inflammatory, *etc*. (Guo *et al.*, 2007 ▸; Przybylski *et al.*, 2009 ▸; Annapoorani & Krishnan, 2013 ▸). A full range of metal complexes formed with these usually multidentate ligands often result in species with enhanced biological action (Bagihalli *et al.*, 2008 ▸; Tian *et al.*, 2009 ▸, 2011 ▸; Chohan *et al.*, 2001 ▸). As part of our ongoing studies of Schiff base ligands and their metal complexes (Manawar, Gondaliya, Mamtora *et al.*, 2019 ▸), the crystal and mol­ecular structures, Hirshfeld surface analysis and computational study of homoleptic Co^II^ (I) and Cu^II^ (II) complexes derived from 2-{(1*E*)-[(*E*)-2-(2,6-di­chloro­benzyl­idene)hydra­zin-1-yl­idene]meth­yl}phenol (Manawar, Gondaliya, Shah *et al.*, 2019 ▸) are described herein.
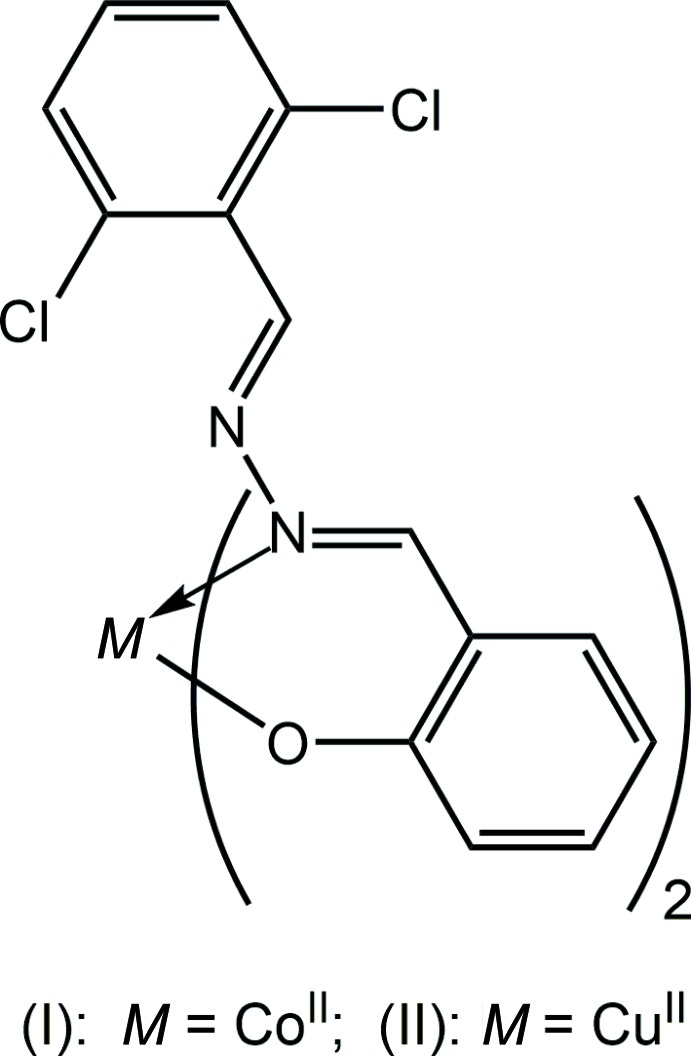


## Structural commentary   

**Figure 1:**
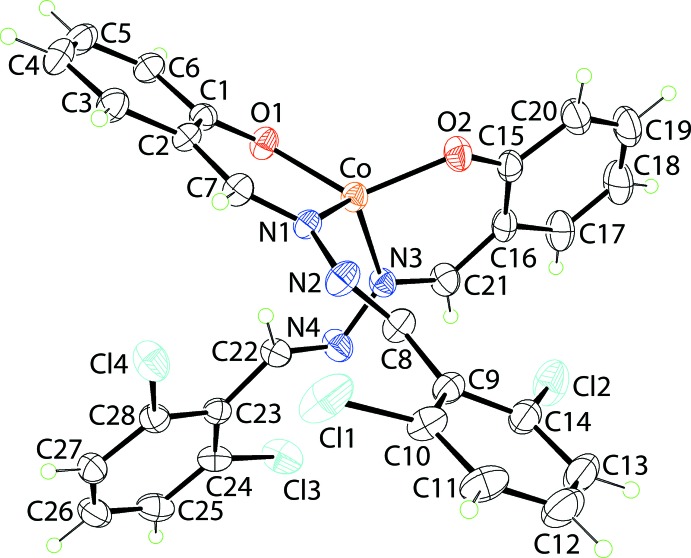
The mol­ecular structure of (I) showing the atom-labelling scheme and displacement ellipsoids at the 35% probability level.

The cobalt complex (I), Fig. 1 ▸, lacks crystallographic symmetry and the metal ion is *N*,*O*-coordinated by two mono-anionic Schiff base ligands; selected geometric parameters are collated in Table 1 ▸. The N_2_O_2_ donor set defines an approximate tetra­hedron with the range of tetra­hedral angles being over 30°. Thus, the narrowest angle of 94.06 (7)° is found for O1—Co—N1 while the widest of 125.33 (8)° is noted for O1—Co—O2. A geometric measure of a four-coordinate geometry is the value of τ_4_, which has values of τ_4_ = 1.0 for an ideal tetra­hedron and τ_4_ = 0.0 for an ideal square-planar geometry (Yang *et al.*, 2007 ▸). In (I), τ_4_ = 0.82, indicating a geometry close to trigonal pyramidal. Each of the Schiff base ligands forms a six-membered Co,O,C_3_,N chelate ring. These adopt an envelope conformation with the Co atom lying 0.253 (3) Å out of the least-squares plane defined by the five remaining atoms of the O1-chelate ring (r.m.s. deviation = 0.0086 Å); the equivalent values for the O2-chelate ring are 0.376 (3) and 0.0222 Å, respectively. The dihedral angle formed between the planar regions of the chelate rings is 86.44 (8)°, consistent with a near to orthogonal relationship. For the O1-chelate ring, the dihedral angle between the five co-planar atoms and the fused-benzene and pendent di­chloro­benzene rings are 0.92 (13) and 7.34 (14)°, respectively, and the dihedral angle between the benzene rings is 6.47 (15)°, indicating a small deviation from planarity. The equivalent dihedral angles for the O2-chelate ring are 1.99 (14), 7.25 (12) and 5.58 (12)°, respectively. These data are consistent with small twists about the N1—N2 [the C7—N1—N2—C8 torsion angle = 166.6 (2)°] and C16—C21 [C15—C16—C21—N3 = 6.4 (4)°] bonds. Finally, each Schiff base ligand features two imine bonds, with the bond length involving the coordinated N1 atom [1.304 (3) Å] being longer than the second N2-imine bond [1.251 (3) Å]; for the O2-ligand, C21—N3 = 1.303 (3) Å and C22—N4 = 1.247 (3) Å. The configurations about imine bonds are different with those involving the coordinated N1 and N3 atoms being *Z*, while the configurations about the other imine bonds are *E*.

Recently, the crystal structure of the precursor Schiff base ligand became available (Manawar, Gondaliya, Shah *et al.*, 2019 ▸). Here, each imine bond has an *E*-configuration and the bond lengths of the imine bonds, *i.e*. 1.281 (2) and 1.258 (3) Å, are inter­mediate to those noted in (I). A very similar conformation of the Schiff base ligand is found with a small twist about the central N—N bond [the C—N—N—C torsion angle = −172.7 (2)°]. The dihedral angles between the outer rings is 4.83 (13)°.

**Figure 2:**
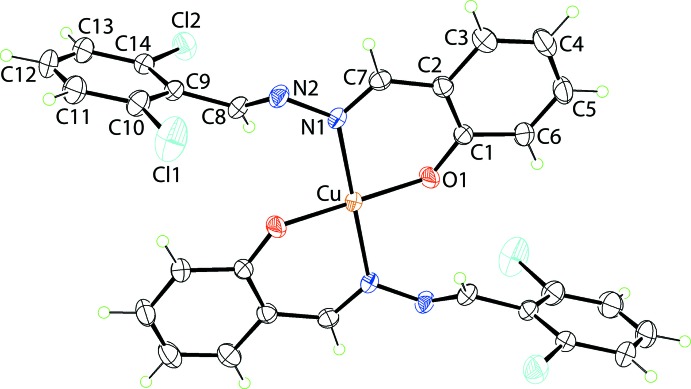
The mol­ecular structure of (II) showing the atom-labelling scheme and displacement ellipsoids at the 35% probability level. Unlabelled atoms are related by the symmetry operation 1 − *x*, 1 − *y*, 1 − *z*.

A distinct coordination geometry is found in the Cu^II^ complex, (II), Fig. 2 ▸ and Table 1 ▸. The Cu^II^ atom lies on a crystallographic centre of inversion. As for (I), *N*,*O*-chelation is observed. From symmetry, the N_2_O_2_ donor set is strictly planar. The Cu^II^ atom lies 0.2582 (13) Å above the resultant square-plane. The chelate rings adopt an envelope conformation, as for (I), with the Cu atom lying 0.470 (2) Å above the plane through the remaining atoms of the chelate ring (r.m.s. deviation = 0.0129 Å). The magnitude of the dihedral angle between the five co-planar atoms of the chelate ring and the fused-benzene ring [1.43 (13)°] resembles the situation in (I), but that formed with pendent di­chloro­benzene ring is quite distinct, at 82.63 (8)°, consistent with an orthogonal relationship. This is reflected in the C7—N1—N2—C8 torsion angle of 141.33 (19)°. The different configuration arises to avoid steric hindrance within the square-planar environment. The Cu—O,N bond lengths span a wider range, *i.e*. 0.14 Å, *c.f.* 0.11 Å for the Co—O,N bond lengths in (II), with the Cu—O bond lengths being shorter than the Co—O bonds, and the Cu—N bonds being longer than the Co—N bonds. Comparable trends are seen in the configurations of the imine bonds, Table 1 ▸.

## Supra­molecular features   

**Figure 3:**
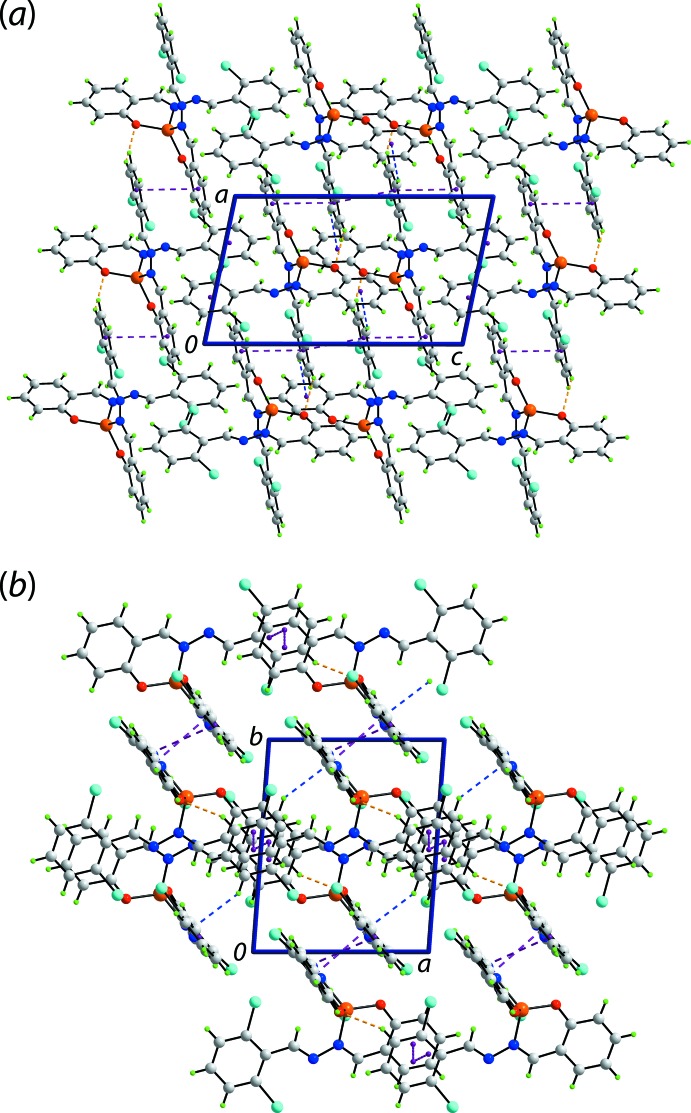
Mol­ecular packing in the crystal of (I): (*a*) supra­molecular layer sustained by C—H⋯O, C—H⋯π and π–π inter­actions shown as orange, blue and purple dashed lines, respectively, and (*b*) a view of the unit-cell contents in a projection down the *c* axis.

The geometric parameters characterizing a number of the identified inter­molecular contacts in the crystal of (I) are listed in Table 2 ▸. Globally, the mol­ecular packing can be described as comprising inter-digitated layers stacked along the the *b*-axis direction. Thus, layers in the *ac* plane are consolidated by chloro­benzene-C—H⋯O(coordinated), chloro­benzene-C—H⋯π(fused-benzene ring) as well as π(fused-benzene, chloro­benzene)–π(chloro­benzene) inter­actions [*Cg*(C15–C20)⋯*Cg*(C23–C28) separation = 3.6460 (17) Å with angle of inclination = 5.57 (13)° for symmetry operation −1 + *x*, *y*, *z* and *Cg*(C23–C28)⋯*Cg*(C23–C28) = 3.6580 (16) Å with angle of inclination = 0° for symmetry operation 2 − *x*, 1 − *y*, 1 − *z*]; the specified π–π inter­actions involve rings derived from the O2-ligand only. A view of the supra­molecular layer is shown in Fig. 3 ▸(*a*). As highlighted in Fig. 3 ▸(*b*), the layers inter-digitate along the *b*-axis. The connections between layers are chloro­benzene-C—H⋯π(fused-benzene ring) as well as π–π inter­actions (involving rings of the O1-ligand only) between centrosymmetrically related fused-benzene rings [π(C1–C6)⋯π(C1–C6) = 3.6916 (16) Å for symmetry operation 1 − *x*, − *y*, 1 − *z* and π(chloro­benzene)–π(chloro­benzene) rings = 3.7968 (19) Å for symmetry operation 1 − *x*, − *y*, 2 − *z*].

**Figure 4:**
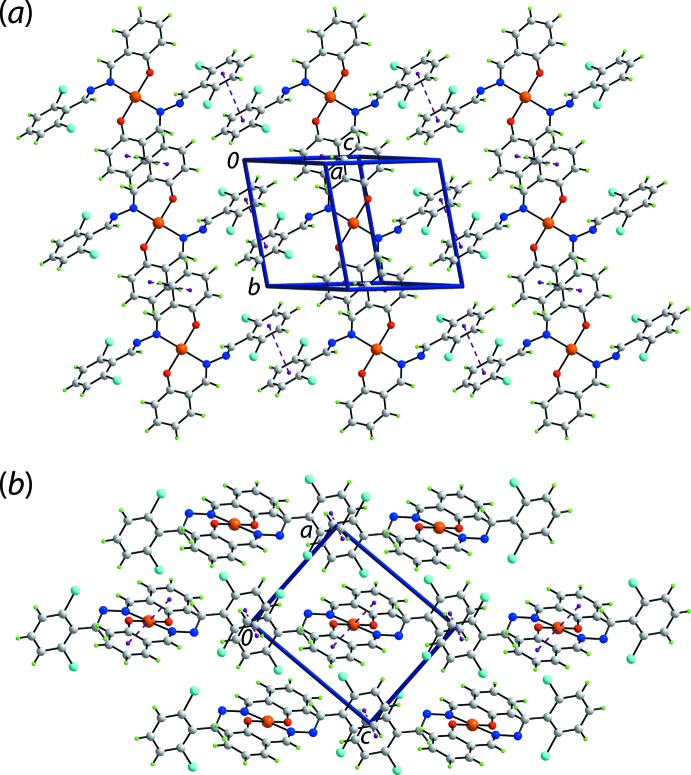
Mol­ecular packing in the crystal of (II): (*a*) supra­molecular layer sustained by π–π inter­actions shown as purple dashed lines and (*b*) a view of the unit-cell contents in a projection down the *b* axis.

The key feature of the mol­ecular packing in the crystal of (II) is the formation of π–π inter­actions between centrosymmetrically related mol­ecules. To a first approximation, the mol­ecular packing resembles that of (I) in that layers assemble into a three-dimensional architecture. As seen in Fig. 4 ▸(*a*), the layers are flat and are sustained by π(fused-benzene)–π(fused-benzene) [inter-centroid *Cg*(C1–C6)⋯*Cg*(C1—C6) separation = 3.8889 (15) Å for symmetry operation 1 − *x*, − *y*, 1 − *z*] and π(di­chloro­benzene)—π(di­chloro­benzene) [inter-centroid separation = *Cg*(C9–C14)⋯*Cg*(C9—C14) = 3.8889 (15) Å for symmetry operation − *x*, 1 − *y*, − *z*] inter­actions. The layers lie parallel to (10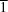) and stack without directional inter­actions between them. A view of the stacking of layers/unit-cell contents is shown in Fig. 4 ▸(*b*).

## Hirshfeld surface analysis   

The Hirshfeld surfaces were calculated for each of (I) and (II) employing *Crystal Explorer 17* (Turner *et al.*, 2017 ▸) and literature protocols (Tan *et al.*, 2019 ▸). This study was undertaken in order to determine the influence of weak, non-covalent inter­actions upon the mol­ecular packing in the absence of conventional hydrogen bonding.

**Figure 5:**
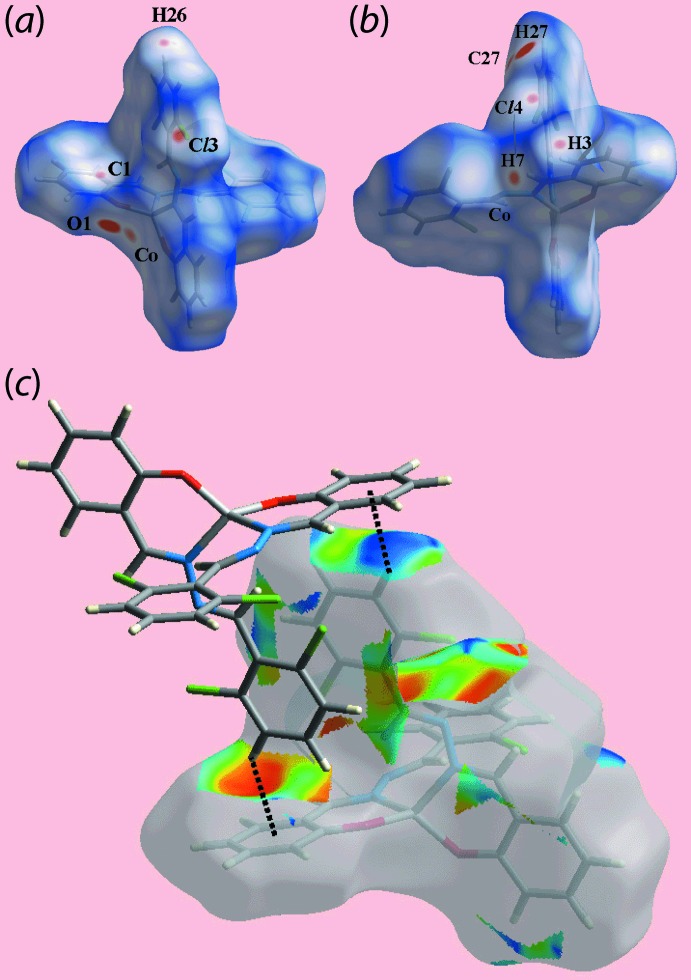
View of the Hirshfeld surface for (I) mapped (*a*) and (*b*) over *d*_norm_ in the range −0.123 to + 1.343 arbitrary units and (*c*) with the shape-index property highlighting inter­molecular C—H⋯π/π⋯H—C contacts.

**Figure 6:**
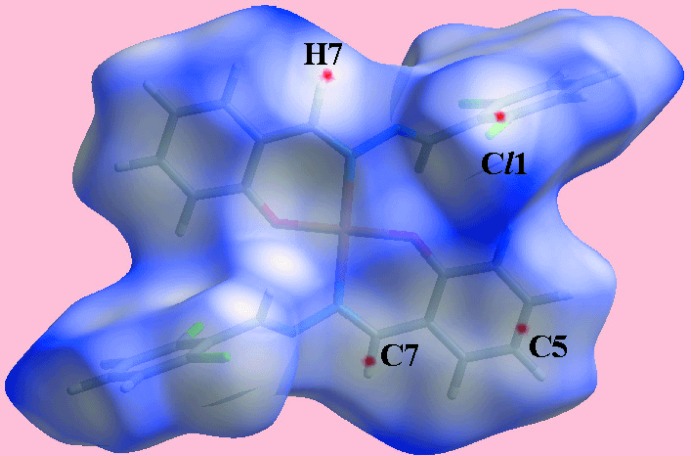
A view of the Hirshfeld surface for (II) mapped over *d*_norm_ in the range −0.016 to 1.528 arbitrary units.

On the Hirshfeld surface mapped over *d*_norm_ for (I) in Fig. 5 ▸(*a*) and (*b*), the bright-red spots near the H27 atom of the (C23–C28) ring and the coordinating O1 atom are an indication of the C—H⋯O inter­action. Referring to Table 3 ▸, the presence of short inter­atomic contacts involving the Co^II^, chloride and chloro­benzene-hydrogen atoms and the atoms of the C1–C6 benzene ring are characterized as faint-red spots near the respective atoms on the *d*_norm_-mapped Hirshfeld surface. The blue bump near the H25 atom and the bright-orange spot about the C1–C6 ring on the Hirshfeld surface mapped with shape-index property in Fig. 5 ▸(*c*) correspond to the donor and acceptor of the C—H⋯π contact. The absence of strong, directional inter­actions in the crystal structure of (II) is evident from its Hirshfeld surface mapped over *d*_norm_ in Fig. 6 ▸, as the surface contains only some tiny, diffuse red spots near the atoms corresponding to short inter­atomic Cl⋯H and C⋯C contacts listed in Table 4 ▸.

**Figure 7:**
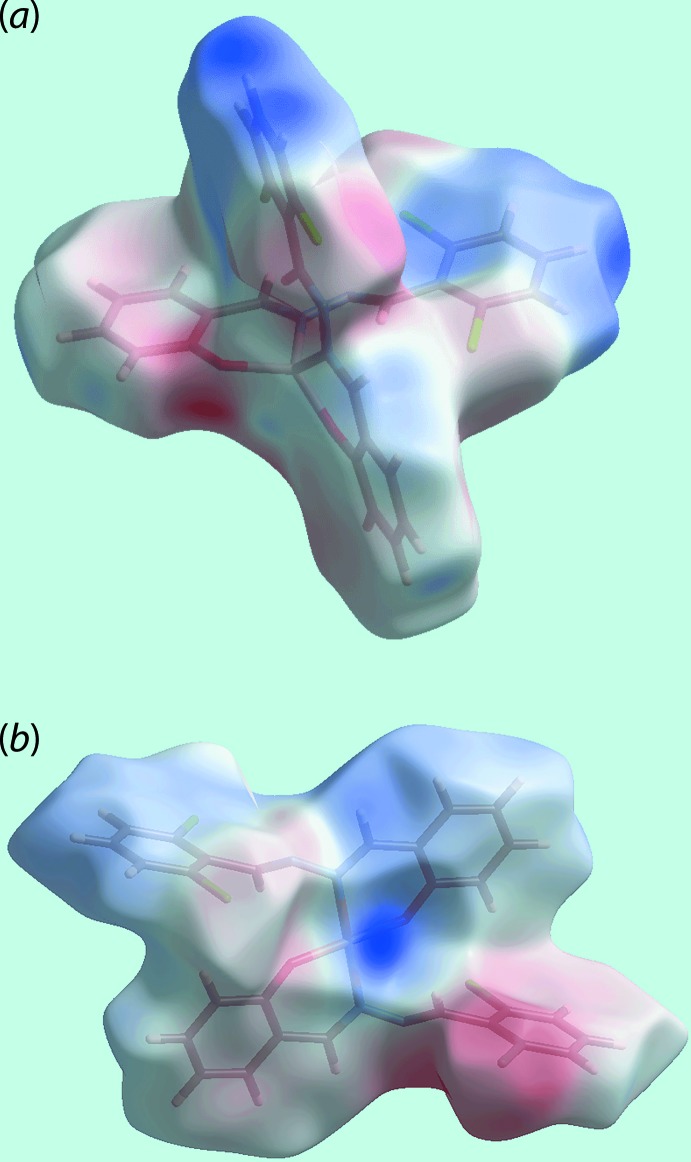
Views of the Hirshfeld surfaces mapped over the electrostatic potential (the red and blue regions represent negative and positive electrostatic potentials, respectively) for (*a*) (I) in the range −0.084 to +0.061 atomic units and (*b*) (II) in the range −0.095 to +0.163 atomic units.

**Figure 8:**
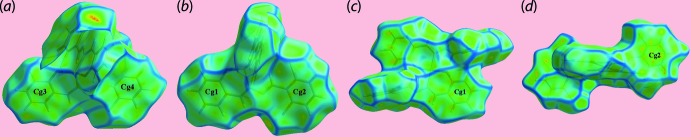
Views of Hirshfeld surfaces mapped over curvedness for (*a*) and (*b*) (I), and (*c*) and (*d*) for (II). The flat regions about aromatic constituents labelled with *Cg*(1)–*Cg*(4) for (I), and *Cg*(1) and *Cg*(2) for (II) indicate the involvement of these rings in π–π stacking inter­actions

On the Hirshfeld surfaces mapped over the electrostatic potential for (I) in Fig. 7 ▸(*a*), the donors and acceptors of the C—H⋯O and C—H⋯π contacts (Table 3 ▸) are viewed as blue and red regions near the respective atoms corresponding to positive and negative electrostatic potentials. The presence of a blue region near the Cu^II^ atom and red region near the Cl2 atom in the corresponding surface for (II) in Fig. 7 ▸(*b*) is an indication of a short inter­molecular Cu⋯Cl2 inter­action [3.2858 (7) Å], as discussed further below, see *Computational chemistry*. The influence of π–π stacking inter­actions in each of the crystals of (I) and (II) is evident as the flat regions about the participating aromatic rings on the Hirshfeld surfaces mapped over curvedness illustrated in Fig. 8 ▸(*a*)–(*d*).

**Figure 9:**
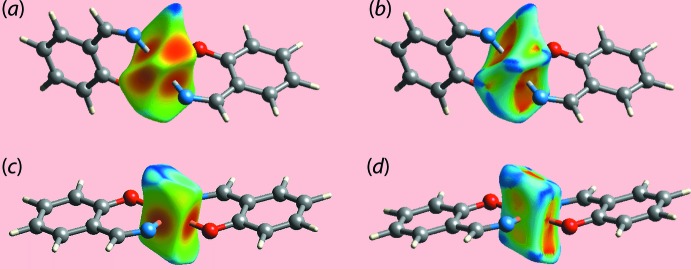
Views of the Hirshfeld surfaces calculated for the Co^II^ (I) and Cu^II^ (II) centres alone, highlighting the coordination geometries formed by the N_2_O_2_ donor sets mapped over (*a*) the distance *d*_e_ external to the surface in the range 0.922 to 2.221 Å for (I), (*b*) the shape-index (S) from −1.0 to +1.0 (arbitrary units) for (I), (*c*) the distance *d*_e_ external to the surface in the range 0.919 to 2.114 Å for (II) and (*d*) the shape-index (S) from −1.0 to +1.0 (arbitrary units) for (II).

Given the different coordination geometries in (I) and (II), it was thought of inter­est to calculate the Hirshfeld surfaces about the individual metal centres (Pinto *et al.*, 2019 ▸). The different coordination geometries, approximately trigonal pyramidal for Co^II^, Fig. 9 ▸(*a*) and (*b*), and square-planar for Cu^II^ in Fig. 9 ▸(*c*) and (*d*), are clearly evident from the illus­trated surfaces although the *M*—O and *M*—N bond lengths are similar, at least to a first approximation, in (I) and (II), Table 1 ▸.

**Figure 10:**
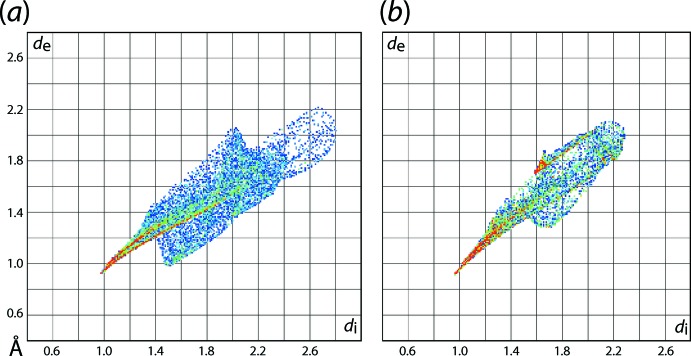
The two-dimensional fingerprint plots taking into account only the Hirshfeld surface about the metal centre in (*a*) (I) and (*b*) (II).

The different coordination geometries about the metal centres are also reflected in the two-dimensional fingerprint plots shown in Fig. 10 ▸, only taking into account the Hirshfeld surface about the metal atom. The distribution of aligned red points from *d*_e_ + *d*_i_ ∼1.8 Å (lower portion) and *d*_e_ + *d*_i_ ∼2.0 Å (upper portion) for the Co—O and Co—N bonds, respectively, in (I) show different inclinations, Fig. 10 ▸(*a*), whereas the superimposed red points in the case of (II), Fig. 10 ▸(*b*), arise as a result of the symmetrical coordination geometry. For (I), the presence of short intra­molecular Co⋯H contacts formed with the chloro­benzene-H8 and H22 atoms (Co⋯H8 = 2.64 Å and Co⋯H22 = 2.55 Å) result in dissymmetry in the Hirshfeld surface and are characterized as bright-orange spots on the shape-index-mapped surface in Fig. 9 ▸(*a*). The square-planar coordination geometry formed by the N_2_O_2_ donor set in (II) results in an approximate cuboid Hirshfeld surface with rounded corners and edges.

**Figure 11:**
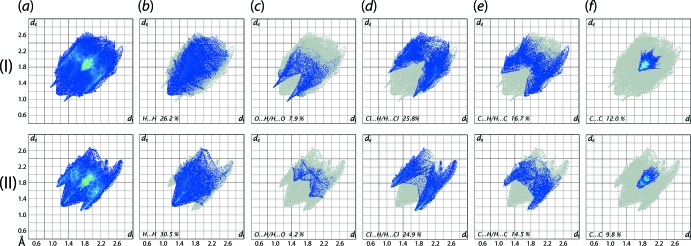
(*a*) A comparison of the full two-dimensional fingerprint plot for each of (I) and (II) and those delineated into (*b*) H⋯H, (*c*) O⋯H/H⋯O, (*d*) Cl⋯H/H⋯Cl, (*e*) C⋯H/H⋯C and (*f*) C⋯C contacts.

The overall two-dimensional fingerprint plots for (I) and (II) are shown in Fig. 11 ▸(*a*), and those delineated into H⋯H, O⋯H/H⋯O, Cl⋯H/H⋯Cl, C⋯H/H⋯C and C⋯C contacts are illustrated in Fig. 11 ▸(*b*)–11(*f*), respectively. The percentage contributions from different inter­atomic contacts to the Hirshfeld surfaces of (I) and (II) are summarized in Table 4 ▸. The presence of chloride in both crystals, and their participation in inter­molecular contacts, has decreased the percentage contributions from H⋯H contacts to the respective Hirshfeld surfaces, Table 4 ▸.

In the fingerprint plot delineated into H⋯H contacts of Fig. 11 ▸(*b*), the short inter­atomic contacts result in a peak at *d*_e_ + *d*_i_ ∼2.3 Å in the crystal of (I) while H⋯H in (II) are at van der Waals separations or longer. The presence of the C—H⋯O contact in (I) is recognized as the pair of spikes at *d*_e_ + *d*_i_ ∼2.2 Å in the fingerprint plot delineated into O⋯H/H⋯O contacts of Fig. 11 ▸(*c*) whereas the comparatively small contribution from these contacts in (II), Table 4 ▸, show the points farther than sum of their van der Waals radii. The pair of forceps-like tips at *d*_e_ + *d*_i_ ∼2.8 Å in the fingerprint plots delineated into Cl⋯H/H⋯Cl contacts in Fig. 11 ▸(*d*) for each of (I) and (II) reflect the presence of Cl⋯H contacts in their crystals; for (II), these are beyond the sum of the van der Waals radii. From the fingerprint plot delineated into C⋯H/H⋯C contacts, Fig. 11 ▸(*e*), the pair of short tips at *d*_e_ + *d*_i_ ∼2.7 Å indicate the presence of C⋯H and C—H⋯π contacts in (I), by contrast to only van der Waals contacts in (II). In the fingerprint plot delineated into C⋯C contacts for (I) and (II), Fig. 11 ▸(*f*), the influence of π–π stacking inter­actions are characterized as the distribution of green points in the plot around *d*_e_ = *d*_i_ = 1.8 Å.

**Figure 12:**
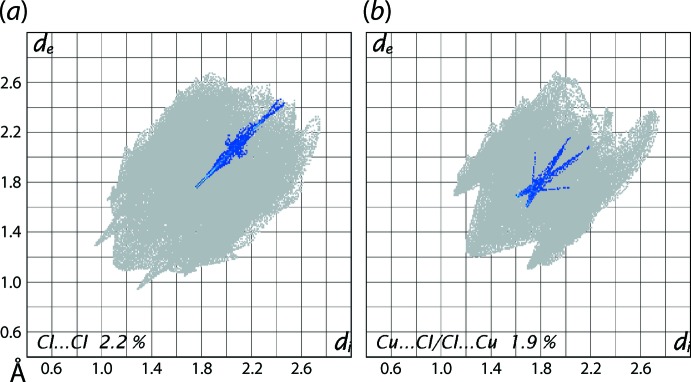
The fingerprint plot delineated into (*a*) Cl⋯Cl contacts for (I) and (*b*) Cu⋯Cl/Cl⋯Cu contacts for (II).

Referring to Fig. 12 ▸(*a*), the distribution of points in the form of a thin line from *d*_e_ + *d*_i_ ∼3.7 Å in the fingerprint plot delineated into Cl⋯Cl contacts for (I) is an indication of influence of these contacts on the packing of (I); this is confirmed in the next section, *Computational study*. The fingerprint plot delineated into Cu⋯Cl/Cl⋯Cu contacts of Fig. 12 ▸(*b*), with the small, *i.e.* 1.9%, but important contribution to the Hirshfeld surface of (II) is the result of a Cu⋯Cl inter­action prominent in its mol­ecular packing, as justified from the inter­action energy calculations described in the next section.

## Computational chemistry   

The pairwise inter­action energies between the mol­ecules in the crystals of each of (I) and (II) were calculated by summing up four energy components, comprising electrostatic (*E*_ele_), polarization (*E*_pol_), dispersion (*E*_dis_) and exchange-repulsion (*E*_rep_) as per the literature (Turner *et al.*, 2017 ▸). In the present study, the energies were obtained by using the wave function calculated at the HF/3-21G level of theory. The nature and the strength of the energies for the key identified inter­molecular inter­actions are qu­anti­tatively summarized in Tables 5 ▸ and 6 ▸ for (I) and (II), respectively.

For (I), among the inter­molecular energies listed in Table 5 ▸, the atoms of (C23–C28) ring involved in the short inter­atomic C⋯H/H⋯C contacts, inter­molecular C—H⋯π and π–π stacking inter­actions between the same pair of symmetry-related mol­ecules have maximum inter­action energies. The dispersive component makes a major contribution to all of the inter­molecular inter­actions in the crystal of (I). The low inter­action energies for Cl⋯H and Cl⋯Cl contacts are consistent with the small contributions from these contacts in the crystal. The presence of a Cu⋯Cl2 contact in the crystal of (II) is an important feature of the packing. This inter­action shows maximum inter­action energy (Table 6 ▸) with significant contributions from the electrostatic component compared to π–π stacking and other inter­molecular inter­actions influential in the mol­ecular packing.

**Figure 13:**
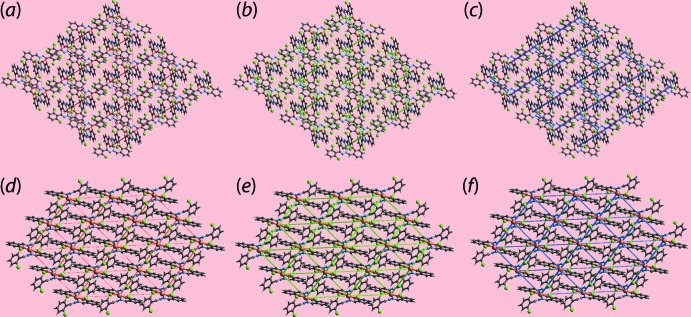
The energy frameworks calculated for (I) showing the (*a*) electrostatic potential force, (*b*) dispersion force and (*c*) total energy; the equivalent diagrams for (II) are shown in (*d*)–(*f*). The energy frameworks were adjusted to the same to same scale factor of 30 with a cut-off value of 3 kJ mol^−1^ within 2 × 2 × 2 unit cells.

The magnitudes of inter­molecular energies are represented graphically in the energy framework diagrams of Fig. 13 ▸(*a*)–(*f*). Here, the supra­molecular architecture of each crystal is visualized through cylinders joining the centroids of mol­ecular pairs using a red, green and blue colour scheme, representing the *E*_ele_, *E*_disp_ and *E*_tot_ components, respectively; the stronger the inter­action, the thicker the cylinder.

## Database survey   

Schiff bases related to those reported in (I) and (II), *i.e*. having two imine functionalities and a single phenol/phenoxide atom/ion on one ring only are quite rare. Thus, the only structure of an analogue available for comparison is a *N*,*O*-chelated di­methyl­aluminium compound with the ring bearing the phenoxide-oxygen also carrying *t*-butyl groups at the 3,5 positions and the second benzene ring bearing a chlorine atom in the 4-position (UPEKEH; Hsu *et al.*, 2017 ▸). By contrast, there are numerous examples of coordination complexes derived from Schiff base mol­ecules with two 2-phenol substituents in each ring, *L*H_2_. In these instances, the dinegative Schiff base anion *N*,*O*-chelates two metal centres such as in binuclear Co_2_(*L* ^1^)_3_ (JUKZOG; Liu *et al.*, 2015 ▸), with a *fac*-N_3_O_3_ donor set within an octa­hedral geometry, and Cu_2_(*L* ^2^)_3_(PPh_3_)_2_ (VOWBAM; Prakash *et al.*, 2015 ▸) with tetra­hedral NOP_2_ donor sets; for the *L* ^1^ dianion, there are 3-eth­oxy substituents in each ring and for *L* ^2^, the are 4-chloro substituents.

## Synthesis and crystallization   

The title complexes (I) and (II) were synthesized according to the literature procedure (Manawar, Gondaliya, Mamtora *et al.*, 2019 ▸). Briefly, the complexes were obtained by mixing the Schiff base, in ethanol, with an aqueous solution of the corresponding metal chloride in 1:1 and 1:2 molar ratios, respectively, in the presence of piperidine as basic catalyst for proton abstraction from the ligand mol­ecules. The crystals in the form of red (I) and dark-brown (II) blocks were grown by slow evaporation from their respective chloro­form solutions.

## Refinement   

Crystal data, data collection and structure refinement details are summarized in Table 7 ▸. The carbon-bound H atoms were placed in calculated positions (C—H = 0.93 Å) and were included in the refinement in the riding model approximation, with *U*_iso_(H) set to 1.2*U*_eq_(C).

## Supplementary Material

Crystal structure: contains datablock(s) I, II, global. DOI: 10.1107/S2056989019016529/hb7872sup1.cif

Structure factors: contains datablock(s) I. DOI: 10.1107/S2056989019016529/hb7872Isup2.hkl

Structure factors: contains datablock(s) II. DOI: 10.1107/S2056989019016529/hb7872IIsup3.hkl

CCDC references: 1891529, 1970822

Additional supporting information:  crystallographic information; 3D view; checkCIF report

## Figures and Tables

**Table 1:** Selected geometric parameters (Å, °) for (I) and (II)*^*a*^*

Parameter	(I): *M* = Co^II^	(II): *M* = Cu^II^
M—O1	1.8940 (17)	1.8776 (14)
M—O2	1.8937 (17)	1.8776 (14)*^*a*^*
M—N1	1.9988 (19)	2.0211 (16)
M—N3	1.999 (2)	2.0211 (16)*^*a*^*
N1—N2	1.411 (3)	1.416 (2)
N3—N4	1.410 (3)	1.416 (2)*^*a*^*
C7—N1	1.304 (3)	1.294 (2)
C8—N2	1.251 (3)	1.258 (3)
C21—N3	1.303 (3)	1.294 (2)*^*a*^*
C22—N4	1.247 (3)	1.258 (3)*^*a*^*
		
O1—Co—O2	125.33 (8)	180*^*a*^*
O1—Co—N1	94.06 (7)	90.28 (6)
O1—Co—N3	112.12 (8)	89.72 (6)
O2—Co—N1	113.82 (8)	89.72 (6)*^*a*^*
O2—Co—N3	94.60 (8)	90.28 (6)*^*a*^*
N1—Co—N3	119.03 (8)	180*^*a*^*

Notes: (*a*) The Cu^II^ atom in (II) lies on a centre of inversion so O2 is equivalent to O1, N3 to N1, *etc.* and are related by the symmetry operation 1 − *x*, 1 − *y*, 1 − *z*.

**Table 2:** Hydrogen-bond geometry (Å, °) for (I) *Cg*3 is the centroid of the C1–C6 ring.

*D*—H⋯*A*	*D*—H	H⋯*A*	*D*⋯*A*	*D*—H⋯*A*
C27—H27⋯O1^i^	0.93	2.35	3.114 (3)	140
C25—H25⋯*Cg*3^ii^	0.93	2.86	3.647 (3)	143

Symmetry codes: (i) 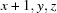; (ii) 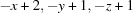.

**Table 3:** Summary of short inter­atomic contacts (Å) in (I) and (II)*^*a*^*

Contact	Distance	Symmetry operation
(I)		
Cl1⋯Cl3	3.4993 (13)	*x*, −1 + *y*, *z*
Cl3⋯H7	2.70	*x*, 1 + *y*, *z*
Cl4⋯H3	2.74	2 − *x*, − *y*, 1 − *z*
C1⋯H26	2.71	2 − *x*, 1 − *y*, 1 − *z*
C6⋯H26	2.76	2 − *x*, 1 − *y*, 1 − *z*
Co⋯C27	3.558 (3)	−1 + *x*, *y*, *z*
Co⋯H27	3.08	−1 + *x*, *y*, *z*
H5⋯H12	2.23	*x*, *y*, −1 + *z*
H5⋯H13	2.30	*x*, *y*, −1 + *z*
(II)		
Cl1⋯H7	2.81	1 − *x*, 1 − *y*, −*z*
C5⋯C7	3.378 (3)	1 − *x*, −*y*, 1 − *z*
Cu⋯Cl2	3.2858 (7)	1 + *x*, *y*, *z*

Notes: (*a*) The inter­atomic distances are calculated in *Crystal Explorer 17* (Turner *et al.*, 2017 ▸) whereby the *X*—H bond lengths are adjusted to their neutron values.

**Table 4:** Percentage contributions of inter­atomic contacts to the Hirshfeld surface for (I) and (II)

Contact	Percentage contribution	
	(I)	(II)
H⋯H	26.2	30.5
O⋯H/H⋯O	7.9	4.2
C⋯H/H⋯C	16.7	14.5
Cl⋯H/H⋯Cl	25.8	24.9
C⋯C	12.0	9.8
N⋯H/H⋯ N	5.5	6.2
Cl⋯Cl	2.2	0.4
C⋯O/O⋯C	0.5	0.3
C⋯N/N⋯C	0.5	1.3
Cl⋯O/O⋯Cl	0.2	0.4
C⋯Cl/Cl⋯C	1.9	3.9
Cl⋯N/N⋯Cl	0.2	1.6
*M*⋯H/H⋯*M*	0.4	0.1
*M*⋯Cl/Cl⋯*M*	0.0	1.9

**Table 5:** Summary of inter­action energies (kJ mol^−1^) calculated for (I)

Contact	*R* (Å)	*E*_ele_	*E*_pol_	*E*_dis_	*E*_rep_	*E*_tot_
H27⋯Co^i^ +	8.81	−21.7	−6.7	−71.3	41.6	−57.0
C27⋯Co^i^ +						
C27—H27⋯O1^i^ +						
*Cg*(C15–C20)⋯*Cg*(C23–C28)^i^						
*Cg*(C9–C14) ⋯*Cg*(C9–C14)^ii^	9.60	−29.6	−7.6	−71.9	32.5	−73.5
*Cg*(C1–C6) ⋯*Cg*(C1–C6)^iii^	10.19	−23.4	−5.5	−58.3	29.4	−56.0
Cl4⋯H3^iv^ +	10.54	−12.7	−1.2	−26.4	19.8	−21.4
Cl1⋯Cl3^iv^						
Cl3⋯H7^v^	10.48	−3.9	−1.3	−14.8	13.4	−7.3
C1⋯H26^vi^ +	9.86	−37.0	−8.0	−84.1	48.4	−79.5
C6⋯H26^vi^ +						
C25–H25⋯*Cg*(C1–C6)^vi^ +						
*Cg*(C23–C28)⋯*Cg*(C23–C28)^vi^						

Symmetry codes: (i) 1 + *x*, *y*, *z*; (ii) 1 − *x*, − *y*, 2 − *z*; (iii) 1 − *x*, − *y*, 1 − *z*; (iv) 2 − *x*, − *y*, 1 − *z*; (v) *x*, 1 + *y*, *z*; (vi) 2 − *x*, 1 − *y*, 1 − *z*.

**Table 6:** Summary of inter­action energies (kJ mol^−1^) calculated for (II)

Contact	*R* (Å)	*E*_ele_	*E*_pol_	*E*_dis_	*E*_rep_	*E*_tot_
*Cg*(C9–C14)⋯*Cg*(C9–C14)^i^	12.94	−0.7	−3.0	−44.5	14.8	−30.8
Cu⋯Cl2^ii^	8.13	−33.0	−5.6	−64.1	44.4	−59.0
Cl1⋯H7^iii^	9.74	−3.7	−2.5	−25.7	15.4	−16.0
C5⋯C7^iv^ +	8.51	−14.4	−4.7	−68.5	36.8	−49.6
*Cg*(C1–C6)⋯*Cg*(C1–C6)^iv^						

Symmetry codes: (i) − *x*, 1 − *y*, − *z*; (ii) 1 + *x*, *y*, *z*; (iii) 1 − *x*, 1 − *y*, −*z*; (iv) 1 − *x*, − *y*, 1 − *z*.

**Table 7:** Experimental details

	(I)	(II)
Crystal data		
Chemical formula	[Co(C_14_H_9_Cl_2_N_2_O)_2_]	[Cu(C_14_H_9_Cl_2_N_2_O)_2_]
*M*_r_	643.19	647.80
Crystal system, space group	Triclinic, *P* 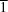	Triclinic, *P* 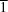
Temperature (K)	296	296
*a*, *b*, *c* (Å)	8.8137 (10), 10.4801 (12), 15.0785 (17)	8.1300 (7), 8.5072 (11), 9.7386 (13)
α, β, γ (°)	85.684 (7), 77.984 (7), 84.965 (7)	83.240 (4), 87.646 (3), 81.533 (4)
*V* (Å^3^)	1354.7 (3)	661.39 (14)
*Z*	2	1
Radiation type	Mo *K*α	Mo *K*α
μ (mm^−1^)	1.06	1.27
Crystal size (mm)	0.35 × 0.30 × 0.30	0.35 × 0.35 × 0.30

Data collection		
Diffractometer	Bruker Kappa APEXII CCD	Bruker Kappa APEXII CCD
Absorption correction	Multi-scan (*SADABS*; Bruker, 2004 ▸)	Multi-scan (*SADABS*; Bruker, 2004 ▸)
*T*_min_, *T*_max_	0.631, 0.746	0.637, 0.714
No. of measured, independent and observed [*I* > 2σ(*I*)] reflections	45690, 6959, 4590	5554, 3090, 2708
*R*_int_	0.102	0.021
(sin θ/λ)_max_ (Å^−1^)	0.678	0.667

Refinement		
*R*[*F* ^2^ > 2σ(*F* ^2^)], *wR*(*F* ^2^), *S*	0.045, 0.112, 1.04	0.033, 0.093, 1.05
No. of reflections	6959	3090
No. of parameters	352	178
H-atom treatment	H-atom parameters constrained	H-atom parameters constrained
Δρ_max_, Δρ_min_ (e Å^−3^)	0.45, −0.50	0.44, −0.46

Computer programs: *APEX2* and *SAINT* (Bruker, 2004 ▸),*SIR92* (Altomare *et al.*, 1994 ▸), *SHELXL2017/1* (Sheldrick, 2015 ▸), *ORTEP-3 for Windows* (Farrugia, 2012 ▸), *DIAMOND* (Brandenburg, 2006 ▸) and *publCIF* (Westrip, 2010 ▸).

## supplementary crystallographic information

### Bis(2-{[(2,6-dichlorobenzylidene)hydrazinylidene]methyl}phenolato)cobalt(II) (I) Crystal data

**Table:**

[Co(C_14_H_9_Cl_2_N_2_O)_2_]	*Z* = 2
*M**_r_* = 643.19	*F*(000) = 650
Triclinic, *P*1	*D*_x_ = 1.577 Mg m^−^^3^
*a* = 8.8137 (10) Å	Mo *K*α radiation, λ = 0.71073 Å
*b* = 10.4801 (12) Å	Cell parameters from 5585 reflections
*c* = 15.0785 (17) Å	θ = 2.3–22.4°
α = 85.684 (7)°	µ = 1.06 mm^−^^1^
β = 77.984 (7)°	*T* = 296 K
γ = 84.965 (7)°	Block, red
*V* = 1354.7 (3) Å^3^	0.35 × 0.30 × 0.30 mm

### Bis(2-{[(2,6-dichlorobenzylidene)hydrazinylidene]methyl}phenolato)cobalt(II) (I) Data collection

**Table:**

Bruker Kappa APEXII CCD diffractometer	4590 reflections with *I* > 2σ(*I*)
Radiation source: X-ray tube	*R*_int_ = 0.102
ω and φ scan	θ_max_ = 28.8°, θ_min_ = 1.4°
Absorption correction: multi-scan (SADABS; Bruker, 2004)	*h* = −11→11
*T*_min_ = 0.631, *T*_max_ = 0.746	*k* = −14→14
45690 measured reflections	*l* = −20→20
6959 independent reflections	

### Bis(2-{[(2,6-dichlorobenzylidene)hydrazinylidene]methyl}phenolato)cobalt(II) (I) Refinement

**Table:**

Refinement on *F*^2^	Primary atom site location: structure-invariant direct methods
Least-squares matrix: full	Secondary atom site location: difference Fourier map
*R*[*F*^2^ > 2σ(*F*^2^)] = 0.045	Hydrogen site location: inferred from neighbouring sites
*wR*(*F*^2^) = 0.112	H-atom parameters constrained
*S* = 1.04	*w* = 1/[σ^2^(*F*_o_^2^) + (0.031*P*)^2^ + 0.0591*P*] where *P* = (*F*_o_^2^ + 2*F*_c_^2^)/3
6959 reflections	(Δ/σ)_max_ = 0.001
352 parameters	Δρ_max_ = 0.45 e Å^−^^3^
0 restraints	Δρ_min_ = −0.50 e Å^−^^3^

### Bis(2-{[(2,6-dichlorobenzylidene)hydrazinylidene]methyl}phenolato)cobalt(II) (I) Special details

**Table:**

Geometry. All esds (except the esd in the dihedral angle between two l.s. planes) are estimated using the full covariance matrix. The cell esds are taken into account individually in the estimation of esds in distances, angles and torsion angles; correlations between esds in cell parameters are only used when they are defined by crystal symmetry. An approximate (isotropic) treatment of cell esds is used for estimating esds involving l.s. planes.

### Bis(2-{[(2,6-dichlorobenzylidene)hydrazinylidene]methyl}phenolato)cobalt(II) (I) Fractional atomic coordinates and isotropic or equivalent isotropic displacement parameters (Å^2^)

**Table:**

	*x*	*y*	*z*	*U*_iso_*/*U*_eq_	
Co	0.44734 (4)	0.27310 (3)	0.68541 (2)	0.03428 (11)	
Cl1	0.88398 (11)	−0.08192 (9)	0.85762 (6)	0.0821 (3)	
Cl2	0.45007 (10)	0.29954 (8)	0.98752 (6)	0.0675 (2)	
Cl3	0.79790 (9)	0.73174 (7)	0.69395 (6)	0.0643 (2)	
Cl4	0.96728 (8)	0.23680 (7)	0.60664 (6)	0.0566 (2)	
O1	0.48493 (19)	0.28424 (16)	0.55684 (12)	0.0386 (4)	
O2	0.25211 (19)	0.24964 (17)	0.76315 (12)	0.0441 (4)	
N1	0.6051 (2)	0.12543 (18)	0.69189 (13)	0.0310 (4)	
N2	0.6554 (2)	0.0714 (2)	0.77036 (14)	0.0411 (5)	
N3	0.4754 (2)	0.43864 (19)	0.73568 (14)	0.0352 (5)	
N4	0.6127 (2)	0.5040 (2)	0.71934 (17)	0.0470 (6)	
C1	0.5601 (3)	0.1984 (2)	0.50173 (16)	0.0320 (5)	
C2	0.6504 (3)	0.0906 (2)	0.53011 (16)	0.0328 (5)	
C3	0.7307 (3)	0.0055 (3)	0.46435 (18)	0.0424 (6)	
H3	0.788970	−0.065835	0.483233	0.051*	
C4	0.7258 (3)	0.0241 (3)	0.37463 (19)	0.0496 (7)	
H4	0.780924	−0.032128	0.332457	0.060*	
C5	0.6353 (3)	0.1305 (3)	0.34809 (19)	0.0488 (7)	
H5	0.629642	0.144282	0.287166	0.059*	
C6	0.5546 (3)	0.2148 (3)	0.40882 (17)	0.0403 (6)	
H6	0.495089	0.284277	0.388505	0.048*	
C7	0.6684 (3)	0.0616 (2)	0.62082 (17)	0.0355 (6)	
H7	0.732456	−0.011148	0.630792	0.043*	
C8	0.6244 (3)	0.1407 (3)	0.83673 (17)	0.0394 (6)	
H8	0.574799	0.221594	0.828883	0.047*	
C9	0.6609 (3)	0.1035 (2)	0.92574 (17)	0.0377 (6)	
C10	0.7686 (3)	0.0031 (3)	0.94344 (19)	0.0467 (7)	
C11	0.7896 (4)	−0.0315 (3)	1.0308 (2)	0.0573 (8)	
H11	0.860799	−0.099067	1.041003	0.069*	
C12	0.7048 (4)	0.0344 (4)	1.1022 (2)	0.0646 (9)	
H12	0.717869	0.010093	1.160909	0.078*	
C13	0.6007 (4)	0.1359 (3)	1.0883 (2)	0.0588 (8)	
H13	0.544591	0.180895	1.136912	0.071*	
C14	0.5809 (3)	0.1697 (3)	1.00133 (19)	0.0448 (7)	
C15	0.1585 (3)	0.3409 (3)	0.80467 (17)	0.0397 (6)	
C16	0.2042 (3)	0.4638 (3)	0.81575 (18)	0.0410 (6)	
C17	0.0932 (3)	0.5554 (3)	0.8613 (2)	0.0575 (8)	
H17	0.123982	0.635619	0.869447	0.069*	
C18	−0.0578 (4)	0.5289 (4)	0.8936 (2)	0.0671 (10)	
H18	−0.130212	0.591163	0.921242	0.081*	
C19	−0.1010 (3)	0.4085 (4)	0.8846 (2)	0.0649 (9)	
H19	−0.203286	0.389479	0.907762	0.078*	
C20	0.0023 (3)	0.3155 (3)	0.84240 (19)	0.0523 (8)	
H20	−0.030665	0.234560	0.838523	0.063*	
C21	0.3586 (3)	0.5031 (3)	0.78469 (19)	0.0440 (7)	
H21	0.377708	0.582955	0.801210	0.053*	
C22	0.7287 (3)	0.4444 (2)	0.67455 (18)	0.0374 (6)	
H22	0.712804	0.364115	0.657169	0.045*	
C23	0.8864 (3)	0.4856 (2)	0.64614 (16)	0.0333 (5)	
C24	0.9300 (3)	0.6101 (2)	0.64938 (19)	0.0414 (6)	
C25	1.0819 (3)	0.6408 (3)	0.6154 (2)	0.0511 (7)	
H25	1.108921	0.724006	0.618188	0.061*	
C26	1.1928 (3)	0.5492 (3)	0.5775 (2)	0.0525 (8)	
H26	1.293927	0.571199	0.553932	0.063*	
C27	1.1554 (3)	0.4260 (3)	0.57441 (18)	0.0453 (7)	
H27	1.230392	0.363795	0.549057	0.054*	
C28	1.0048 (3)	0.3955 (3)	0.60946 (18)	0.0384 (6)	

### Bis(2-{[(2,6-dichlorobenzylidene)hydrazinylidene]methyl}phenolato)cobalt(II) (I) Atomic displacement parameters (Å^2^)

**Table:**

	*U*^11^	*U*^22^	*U*^33^	*U*^12^	*U*^13^	*U*^23^
Co	0.03317 (18)	0.0346 (2)	0.0336 (2)	0.00280 (14)	−0.00453 (14)	−0.00479 (15)
Cl1	0.1017 (7)	0.0828 (6)	0.0679 (6)	0.0502 (5)	−0.0448 (5)	−0.0328 (5)
Cl2	0.0717 (5)	0.0700 (6)	0.0533 (5)	0.0181 (4)	−0.0018 (4)	−0.0142 (4)
Cl3	0.0601 (5)	0.0356 (4)	0.1007 (7)	0.0009 (3)	−0.0221 (4)	−0.0150 (4)
Cl4	0.0460 (4)	0.0380 (4)	0.0810 (6)	0.0041 (3)	−0.0014 (4)	−0.0129 (4)
O1	0.0414 (9)	0.0376 (10)	0.0348 (10)	0.0096 (8)	−0.0074 (8)	−0.0044 (8)
O2	0.0387 (9)	0.0430 (11)	0.0459 (12)	−0.0004 (8)	0.0022 (8)	−0.0058 (9)
N1	0.0356 (10)	0.0300 (11)	0.0274 (11)	−0.0004 (8)	−0.0075 (9)	−0.0003 (9)
N2	0.0546 (13)	0.0363 (13)	0.0329 (13)	0.0050 (10)	−0.0137 (10)	−0.0005 (10)
N3	0.0302 (10)	0.0345 (12)	0.0393 (13)	0.0028 (9)	−0.0046 (9)	−0.0045 (10)
N4	0.0381 (12)	0.0387 (13)	0.0618 (16)	−0.0019 (10)	−0.0005 (11)	−0.0162 (12)
C1	0.0331 (12)	0.0312 (13)	0.0321 (14)	−0.0049 (10)	−0.0069 (10)	−0.0014 (11)
C2	0.0374 (13)	0.0315 (13)	0.0303 (13)	−0.0020 (10)	−0.0076 (10)	−0.0059 (11)
C3	0.0452 (15)	0.0403 (16)	0.0414 (16)	0.0036 (12)	−0.0086 (12)	−0.0087 (13)
C4	0.0596 (17)	0.0513 (18)	0.0366 (16)	0.0003 (14)	−0.0036 (13)	−0.0166 (14)
C5	0.0617 (18)	0.0576 (19)	0.0302 (15)	−0.0106 (15)	−0.0126 (13)	−0.0062 (14)
C6	0.0453 (14)	0.0424 (16)	0.0347 (15)	−0.0019 (12)	−0.0131 (12)	−0.0010 (12)
C7	0.0407 (13)	0.0293 (13)	0.0357 (15)	0.0045 (10)	−0.0085 (11)	−0.0031 (11)
C8	0.0463 (14)	0.0368 (15)	0.0349 (15)	0.0065 (12)	−0.0112 (12)	−0.0034 (12)
C9	0.0461 (14)	0.0375 (15)	0.0306 (14)	−0.0059 (12)	−0.0096 (11)	−0.0010 (12)
C10	0.0574 (17)	0.0446 (17)	0.0423 (17)	0.0006 (13)	−0.0195 (14)	−0.0087 (13)
C11	0.076 (2)	0.0532 (19)	0.0510 (19)	−0.0012 (16)	−0.0361 (17)	0.0022 (16)
C12	0.083 (2)	0.079 (2)	0.0371 (18)	−0.015 (2)	−0.0227 (17)	0.0032 (17)
C13	0.074 (2)	0.070 (2)	0.0328 (17)	−0.0076 (18)	−0.0091 (15)	−0.0092 (16)
C14	0.0535 (16)	0.0464 (17)	0.0349 (15)	−0.0098 (13)	−0.0069 (12)	−0.0034 (13)
C15	0.0344 (13)	0.0524 (17)	0.0295 (14)	−0.0004 (12)	−0.0033 (11)	0.0037 (12)
C16	0.0372 (13)	0.0466 (16)	0.0343 (15)	0.0073 (12)	0.0004 (11)	−0.0030 (12)
C17	0.0498 (17)	0.0564 (19)	0.056 (2)	0.0133 (14)	0.0064 (14)	−0.0076 (16)
C18	0.0467 (17)	0.076 (3)	0.065 (2)	0.0186 (17)	0.0105 (15)	−0.0072 (19)
C19	0.0350 (15)	0.097 (3)	0.055 (2)	0.0029 (17)	0.0038 (14)	0.0023 (19)
C20	0.0389 (15)	0.064 (2)	0.0497 (19)	−0.0041 (14)	−0.0014 (13)	0.0016 (15)
C21	0.0416 (14)	0.0365 (15)	0.0509 (18)	0.0043 (12)	−0.0035 (12)	−0.0081 (13)
C22	0.0348 (13)	0.0298 (14)	0.0482 (16)	0.0003 (10)	−0.0097 (11)	−0.0057 (12)
C23	0.0348 (12)	0.0344 (14)	0.0317 (14)	−0.0014 (10)	−0.0110 (10)	0.0014 (11)
C24	0.0452 (15)	0.0350 (15)	0.0491 (17)	−0.0046 (11)	−0.0215 (13)	0.0001 (12)
C25	0.0534 (17)	0.0454 (17)	0.061 (2)	−0.0170 (14)	−0.0232 (15)	0.0048 (15)
C26	0.0381 (15)	0.068 (2)	0.0531 (19)	−0.0158 (14)	−0.0116 (13)	0.0036 (16)
C27	0.0345 (13)	0.0602 (19)	0.0405 (16)	0.0005 (13)	−0.0077 (12)	−0.0025 (14)
C28	0.0365 (13)	0.0405 (15)	0.0387 (15)	−0.0001 (11)	−0.0114 (11)	0.0014 (12)

### Bis(2-{[(2,6-dichlorobenzylidene)hydrazinylidene]methyl}phenolato)cobalt(II) (I) Geometric parameters (Å, º)

**Table:**

Co—O2	1.8937 (17)	C9—C14	1.406 (4)
Co—O1	1.8940 (17)	C10—C11	1.385 (4)
Co—N1	1.9988 (19)	C11—C12	1.371 (4)
Co—N3	1.999 (2)	C11—H11	0.9300
Cl1—C10	1.723 (3)	C12—C13	1.376 (4)
Cl2—C14	1.733 (3)	C12—H12	0.9300
Cl3—C24	1.725 (3)	C13—C14	1.375 (4)
Cl4—C28	1.729 (3)	C13—H13	0.9300
O1—C1	1.310 (3)	C15—C20	1.416 (3)
O2—C15	1.313 (3)	C15—C16	1.414 (4)
N1—C7	1.304 (3)	C16—C17	1.416 (4)
N1—N2	1.411 (3)	C16—C21	1.431 (3)
N2—C8	1.251 (3)	C17—C18	1.363 (4)
N3—C21	1.303 (3)	C17—H17	0.9300
N3—N4	1.410 (3)	C18—C19	1.374 (5)
N4—C22	1.247 (3)	C18—H18	0.9300
C1—C6	1.409 (3)	C19—C20	1.371 (4)
C1—C2	1.416 (3)	C19—H19	0.9300
C2—C3	1.417 (3)	C20—H20	0.9300
C2—C7	1.417 (3)	C21—H21	0.9300
C3—C4	1.361 (4)	C22—C23	1.461 (3)
C3—H3	0.9300	C22—H22	0.9300
C4—C5	1.396 (4)	C23—C28	1.397 (3)
C4—H4	0.9300	C23—C24	1.399 (3)
C5—C6	1.366 (4)	C24—C25	1.388 (4)
C5—H5	0.9300	C25—C26	1.374 (4)
C6—H6	0.9300	C25—H25	0.9300
C7—H7	0.9300	C26—C27	1.367 (4)
C8—C9	1.461 (3)	C26—H26	0.9300
C8—H8	0.9300	C27—C28	1.379 (3)
C9—C10	1.402 (4)	C27—H27	0.9300
			
O2—Co—O1	125.33 (8)	C11—C12—H12	119.5
O2—Co—N1	113.82 (8)	C13—C12—H12	119.5
O1—Co—N1	94.06 (7)	C14—C13—C12	119.0 (3)
O2—Co—N3	94.60 (8)	C14—C13—H13	120.5
O1—Co—N3	112.12 (8)	C12—C13—H13	120.5
N1—Co—N3	119.03 (8)	C13—C14—C9	122.7 (3)
C1—O1—Co	127.34 (15)	C13—C14—Cl2	117.0 (2)
C15—O2—Co	125.37 (16)	C9—C14—Cl2	120.3 (2)
C7—N1—N2	111.4 (2)	O2—C15—C20	118.7 (3)
C7—N1—Co	121.70 (16)	O2—C15—C16	124.1 (2)
N2—N1—Co	126.78 (15)	C20—C15—C16	117.2 (2)
C8—N2—N1	114.8 (2)	C15—C16—C17	119.4 (2)
C21—N3—N4	112.1 (2)	C15—C16—C21	124.2 (2)
C21—N3—Co	121.09 (17)	C17—C16—C21	116.4 (3)
N4—N3—Co	126.71 (15)	C18—C17—C16	121.6 (3)
C22—N4—N3	114.3 (2)	C18—C17—H17	119.2
O1—C1—C6	118.8 (2)	C16—C17—H17	119.2
O1—C1—C2	123.5 (2)	C17—C18—C19	118.9 (3)
C6—C1—C2	117.7 (2)	C17—C18—H18	120.6
C3—C2—C7	116.8 (2)	C19—C18—H18	120.6
C3—C2—C1	118.9 (2)	C18—C19—C20	121.9 (3)
C7—C2—C1	124.4 (2)	C18—C19—H19	119.0
C4—C3—C2	122.6 (3)	C20—C19—H19	119.0
C4—C3—H3	118.7	C19—C20—C15	120.9 (3)
C2—C3—H3	118.7	C19—C20—H20	119.5
C3—C4—C5	117.7 (3)	C15—C20—H20	119.5
C3—C4—H4	121.1	N3—C21—C16	126.8 (3)
C5—C4—H4	121.1	N3—C21—H21	116.6
C6—C5—C4	122.1 (3)	C16—C21—H21	116.6
C6—C5—H5	119.0	N4—C22—C23	127.7 (2)
C4—C5—H5	119.0	N4—C22—H22	116.1
C5—C6—C1	121.1 (3)	C23—C22—H22	116.1
C5—C6—H6	119.5	C28—C23—C24	116.2 (2)
C1—C6—H6	119.5	C28—C23—C22	118.2 (2)
N1—C7—C2	127.3 (2)	C24—C23—C22	125.6 (2)
N1—C7—H7	116.4	C25—C24—C23	120.9 (3)
C2—C7—H7	116.4	C25—C24—Cl3	117.3 (2)
N2—C8—C9	124.5 (2)	C23—C24—Cl3	121.8 (2)
N2—C8—H8	117.7	C26—C25—C24	120.5 (3)
C9—C8—H8	117.7	C26—C25—H25	119.7
C10—C9—C14	116.0 (2)	C24—C25—H25	119.7
C10—C9—C8	125.2 (2)	C27—C26—C25	120.4 (3)
C14—C9—C8	118.8 (2)	C27—C26—H26	119.8
C11—C10—C9	121.7 (3)	C25—C26—H26	119.8
C11—C10—Cl1	116.5 (2)	C26—C27—C28	118.9 (3)
C9—C10—Cl1	121.8 (2)	C26—C27—H27	120.5
C12—C11—C10	119.7 (3)	C28—C27—H27	120.5
C12—C11—H11	120.2	C27—C28—C23	123.1 (2)
C10—C11—H11	120.2	C27—C28—Cl4	116.4 (2)
C11—C12—C13	121.0 (3)	C23—C28—Cl4	120.48 (19)
			
O2—Co—O1—C1	−108.33 (19)	C12—C13—C14—Cl2	−179.2 (2)
N1—Co—O1—C1	14.8 (2)	C10—C9—C14—C13	−2.7 (4)
N3—Co—O1—C1	138.45 (19)	C8—C9—C14—C13	175.8 (3)
O1—Co—O2—C15	−99.8 (2)	C10—C9—C14—Cl2	177.6 (2)
N1—Co—O2—C15	146.13 (19)	C8—C9—C14—Cl2	−3.9 (3)
N3—Co—O2—C15	21.5 (2)	Co—O2—C15—C20	164.92 (18)
C7—N1—N2—C8	166.6 (2)	Co—O2—C15—C16	−16.2 (4)
Co—N1—N2—C8	−17.3 (3)	O2—C15—C16—C17	179.6 (3)
C21—N3—N4—C22	178.3 (2)	C20—C15—C16—C17	−1.4 (4)
Co—N3—N4—C22	−6.0 (3)	O2—C15—C16—C21	−1.3 (4)
Co—O1—C1—C6	169.84 (16)	C20—C15—C16—C21	177.6 (2)
Co—O1—C1—C2	−11.6 (3)	C15—C16—C17—C18	−1.1 (4)
O1—C1—C2—C3	−178.2 (2)	C21—C16—C17—C18	179.7 (3)
C6—C1—C2—C3	0.3 (3)	C16—C17—C18—C19	2.6 (5)
O1—C1—C2—C7	1.0 (4)	C17—C18—C19—C20	−1.5 (5)
C6—C1—C2—C7	179.5 (2)	C18—C19—C20—C15	−1.2 (5)
C7—C2—C3—C4	−178.5 (2)	O2—C15—C20—C19	−178.5 (3)
C1—C2—C3—C4	0.7 (4)	C16—C15—C20—C19	2.5 (4)
C2—C3—C4—C5	−1.2 (4)	N4—N3—C21—C16	−178.3 (2)
C3—C4—C5—C6	0.6 (4)	Co—N3—C21—C16	5.7 (4)
C4—C5—C6—C1	0.4 (4)	C15—C16—C21—N3	6.4 (4)
O1—C1—C6—C5	177.8 (2)	C17—C16—C21—N3	−174.5 (3)
C2—C1—C6—C5	−0.8 (4)	N3—N4—C22—C23	179.7 (2)
N2—N1—C7—C2	−177.2 (2)	N4—C22—C23—C28	169.5 (3)
Co—N1—C7—C2	6.5 (3)	N4—C22—C23—C24	−12.9 (4)
C3—C2—C7—N1	−179.5 (2)	C28—C23—C24—C25	1.7 (4)
C1—C2—C7—N1	1.3 (4)	C22—C23—C24—C25	−176.0 (2)
N1—N2—C8—C9	178.1 (2)	C28—C23—C24—Cl3	−179.22 (19)
N2—C8—C9—C10	17.7 (4)	C22—C23—C24—Cl3	3.1 (4)
N2—C8—C9—C14	−160.7 (3)	C23—C24—C25—C26	0.1 (4)
C14—C9—C10—C11	2.5 (4)	Cl3—C24—C25—C26	−179.0 (2)
C8—C9—C10—C11	−175.9 (3)	C24—C25—C26—C27	−1.2 (4)
C14—C9—C10—Cl1	−176.4 (2)	C25—C26—C27—C28	0.2 (4)
C8—C9—C10—Cl1	5.2 (4)	C26—C27—C28—C23	1.8 (4)
C9—C10—C11—C12	−0.8 (5)	C26—C27—C28—Cl4	−177.9 (2)
Cl1—C10—C11—C12	178.2 (3)	C24—C23—C28—C27	−2.7 (4)
C10—C11—C12—C13	−1.0 (5)	C22—C23—C28—C27	175.1 (2)
C11—C12—C13—C14	0.9 (5)	C24—C23—C28—Cl4	176.99 (19)
C12—C13—C14—C9	1.1 (4)	C22—C23—C28—Cl4	−5.2 (3)

### Bis(2-{[(2,6-dichlorobenzylidene)hydrazinylidene]methyl}phenolato)cobalt(II) (I) Hydrogen-bond geometry (Å, º)

*Cg*3 is the centroid of the C1–C6 ring.

**Table:**

*D*—H···*A*	*D*—H	H···*A*	*D*···*A*	*D*—H···*A*
C27—H27···O1^i^	0.93	2.35	3.114 (3)	140
C25—H25···*Cg*3^ii^	0.93	2.86	3.647 (3)	143

Symmetry codes: (i) *x*+1, *y*, *z*; (ii) −*x*+2, −*y*+1, −*z*+1.

### Bis(2-{[(2,6-dichlorobenzylidene)hydrazinylidene]methyl}phenolato)copper(II) (II) Crystal data

**Table:**

[Cu(C_14_H_9_Cl_2_N_2_O)_2_]	*Z* = 1
*M**_r_* = 647.80	*F*(000) = 327
Triclinic, *P*1	*D*_x_ = 1.626 Mg m^−^^3^
*a* = 8.1300 (7) Å	Mo *K*α radiation, λ = 0.71073 Å
*b* = 8.5072 (11) Å	Cell parameters from 3126 reflections
*c* = 9.7386 (13) Å	θ = 4.9–56.5°
α = 83.240 (4)°	µ = 1.26 mm^−^^1^
β = 87.646 (3)°	*T* = 296 K
γ = 81.533 (4)°	Block, dark-brown
*V* = 661.39 (14) Å^3^	0.35 × 0.35 × 0.30 mm

### Bis(2-{[(2,6-dichlorobenzylidene)hydrazinylidene]methyl}phenolato)copper(II) (II) Data collection

**Table:**

Bruker Kappa APEXII CCD diffractometer	2708 reflections with *I* > 2σ(*I*)
ω and φ scan	*R*_int_ = 0.021
Absorption correction: multi-scan (SADABS; Bruker, 2004)	θ_max_ = 28.3°, θ_min_ = 3.3°
*T*_min_ = 0.637, *T*_max_ = 0.714	*h* = −5→10
5554 measured reflections	*k* = −11→11
3090 independent reflections	*l* = −12→12

### Bis(2-{[(2,6-dichlorobenzylidene)hydrazinylidene]methyl}phenolato)copper(II) (II) Refinement

**Table:**

Refinement on *F*^2^	Primary atom site location: structure-invariant direct methods
Least-squares matrix: full	Secondary atom site location: difference Fourier map
*R*[*F*^2^ > 2σ(*F*^2^)] = 0.033	Hydrogen site location: inferred from neighbouring sites
*wR*(*F*^2^) = 0.093	H-atom parameters constrained
*S* = 1.05	*w* = 1/[σ^2^(*F*_o_^2^) + (0.0461*P*)^2^ + 0.2934*P*] where *P* = (*F*_o_^2^ + 2*F*_c_^2^)/3
3090 reflections	(Δ/σ)_max_ < 0.001
178 parameters	Δρ_max_ = 0.44 e Å^−^^3^
0 restraints	Δρ_min_ = −0.46 e Å^−^^3^

### Bis(2-{[(2,6-dichlorobenzylidene)hydrazinylidene]methyl}phenolato)copper(II) (II) Special details

**Table:**

Geometry. All esds (except the esd in the dihedral angle between two l.s. planes) are estimated using the full covariance matrix. The cell esds are taken into account individually in the estimation of esds in distances, angles and torsion angles; correlations between esds in cell parameters are only used when they are defined by crystal symmetry. An approximate (isotropic) treatment of cell esds is used for estimating esds involving l.s. planes.

### Bis(2-{[(2,6-dichlorobenzylidene)hydrazinylidene]methyl}phenolato)copper(II) (II) Fractional atomic coordinates and isotropic or equivalent isotropic displacement parameters (Å^2^)

**Table:**

	*x*	*y*	*z*	*U*_iso_*/*U*_eq_	
Cu	0.500000	0.500000	0.500000	0.02898 (11)	
Cl1	0.31832 (11)	0.73971 (12)	0.01621 (8)	0.0830 (3)	
Cl2	−0.14142 (7)	0.45455 (7)	0.33048 (7)	0.05364 (17)	
O1	0.5954 (2)	0.30738 (17)	0.59863 (16)	0.0435 (4)	
N1	0.4436 (2)	0.37945 (19)	0.34549 (16)	0.0293 (3)	
N2	0.3392 (2)	0.4457 (2)	0.23415 (17)	0.0352 (4)	
C1	0.6593 (3)	0.1736 (2)	0.5513 (2)	0.0338 (4)	
C2	0.6301 (3)	0.1364 (2)	0.4182 (2)	0.0325 (4)	
C3	0.7051 (3)	−0.0117 (3)	0.3762 (3)	0.0439 (5)	
H3	0.684470	−0.036821	0.288672	0.053*	
C4	0.8071 (3)	−0.1183 (3)	0.4617 (3)	0.0519 (6)	
H4	0.857619	−0.214233	0.432071	0.062*	
C5	0.8345 (3)	−0.0824 (3)	0.5923 (3)	0.0519 (6)	
H5	0.903270	−0.155502	0.650883	0.062*	
C6	0.7625 (3)	0.0588 (3)	0.6378 (3)	0.0459 (5)	
H6	0.782038	0.079204	0.726963	0.055*	
C7	0.5198 (3)	0.2392 (2)	0.3251 (2)	0.0321 (4)	
H7	0.500996	0.201827	0.242030	0.038*	
C8	0.2031 (2)	0.5217 (2)	0.2710 (2)	0.0327 (4)	
H8	0.179795	0.526076	0.364870	0.039*	
C9	0.0790 (3)	0.6043 (2)	0.1705 (2)	0.0338 (4)	
C10	0.1164 (3)	0.7083 (3)	0.0554 (2)	0.0456 (5)	
C11	−0.0074 (4)	0.7920 (3)	−0.0292 (3)	0.0597 (7)	
H11	0.019998	0.861210	−0.105066	0.072*	
C12	−0.1692 (4)	0.7730 (3)	−0.0015 (3)	0.0676 (9)	
H12	−0.251592	0.830675	−0.058269	0.081*	
C13	−0.2130 (3)	0.6699 (3)	0.1091 (3)	0.0589 (7)	
H13	−0.323355	0.655485	0.126954	0.071*	
C14	−0.0872 (3)	0.5881 (2)	0.1931 (2)	0.0392 (5)	

### Bis(2-{[(2,6-dichlorobenzylidene)hydrazinylidene]methyl}phenolato)copper(II) (II) Atomic displacement parameters (Å^2^)

**Table:**

	*U*^11^	*U*^22^	*U*^33^	*U*^12^	*U*^13^	*U*^23^
Cu	0.02975 (19)	0.02729 (17)	0.02922 (18)	−0.00160 (12)	−0.00773 (12)	−0.00101 (12)
Cl1	0.0648 (5)	0.1185 (7)	0.0567 (4)	−0.0217 (5)	0.0044 (3)	0.0361 (4)
Cl2	0.0335 (3)	0.0511 (3)	0.0743 (4)	−0.0079 (2)	0.0011 (3)	0.0025 (3)
O1	0.0645 (11)	0.0280 (7)	0.0364 (8)	0.0034 (7)	−0.0205 (7)	−0.0033 (6)
N1	0.0274 (8)	0.0338 (8)	0.0264 (7)	−0.0031 (6)	−0.0058 (6)	−0.0014 (6)
N2	0.0360 (9)	0.0415 (9)	0.0273 (8)	−0.0016 (7)	−0.0089 (7)	−0.0033 (7)
C1	0.0346 (11)	0.0265 (9)	0.0404 (10)	−0.0063 (8)	−0.0087 (8)	0.0015 (7)
C2	0.0301 (10)	0.0297 (9)	0.0377 (10)	−0.0056 (7)	0.0007 (8)	−0.0025 (7)
C3	0.0474 (14)	0.0369 (11)	0.0465 (12)	−0.0020 (9)	0.0044 (10)	−0.0079 (9)
C4	0.0484 (14)	0.0331 (11)	0.0702 (17)	0.0059 (10)	0.0038 (12)	−0.0057 (11)
C5	0.0471 (14)	0.0335 (11)	0.0700 (17)	0.0039 (10)	−0.0135 (12)	0.0067 (11)
C6	0.0529 (14)	0.0336 (10)	0.0498 (13)	−0.0026 (9)	−0.0207 (11)	0.0037 (9)
C7	0.0328 (10)	0.0357 (9)	0.0287 (9)	−0.0066 (8)	0.0001 (7)	−0.0061 (7)
C8	0.0304 (10)	0.0414 (10)	0.0268 (9)	−0.0068 (8)	−0.0060 (7)	−0.0016 (7)
C9	0.0350 (11)	0.0352 (10)	0.0317 (10)	−0.0016 (8)	−0.0095 (8)	−0.0068 (8)
C10	0.0534 (15)	0.0507 (13)	0.0318 (10)	−0.0051 (11)	−0.0099 (9)	−0.0014 (9)
C11	0.084 (2)	0.0506 (14)	0.0422 (13)	−0.0036 (13)	−0.0263 (13)	0.0042 (11)
C12	0.077 (2)	0.0517 (15)	0.0713 (18)	0.0100 (14)	−0.0474 (16)	−0.0024 (13)
C13	0.0419 (15)	0.0499 (14)	0.085 (2)	0.0029 (11)	−0.0289 (13)	−0.0106 (13)
C14	0.0370 (12)	0.0330 (10)	0.0482 (12)	−0.0006 (8)	−0.0126 (9)	−0.0088 (9)

### Bis(2-{[(2,6-dichlorobenzylidene)hydrazinylidene]methyl}phenolato)copper(II) (II) Geometric parameters (Å, º)

**Table:**

Cu—O1	1.8776 (14)	C4—H4	0.9300
Cu—O1^i^	1.8776 (14)	C5—C6	1.371 (3)
Cu—N1	2.0211 (16)	C5—H5	0.9300
Cu—N1^i^	2.0211 (16)	C6—H6	0.9300
Cl1—C10	1.722 (3)	C7—H7	0.9300
Cl2—C14	1.737 (2)	C8—C9	1.476 (3)
O1—C1	1.306 (2)	C8—H8	0.9300
N1—C7	1.294 (2)	C9—C14	1.384 (3)
N1—N2	1.416 (2)	C9—C10	1.396 (3)
N2—C8	1.258 (3)	C10—C11	1.385 (3)
C1—C6	1.411 (3)	C11—C12	1.361 (5)
C1—C2	1.407 (3)	C11—H11	0.9300
C2—C3	1.415 (3)	C12—C13	1.376 (5)
C2—C7	1.428 (3)	C12—H12	0.9300
C3—C4	1.365 (3)	C13—C14	1.387 (3)
C3—H3	0.9300	C13—H13	0.9300
C4—C5	1.377 (4)		
			
O1—Cu—O1^i^	180.00 (10)	C5—C6—H6	119.5
O1—Cu—N1	90.28 (6)	C1—C6—H6	119.5
O1^i^—Cu—N1	89.72 (6)	N1—C7—C2	126.18 (18)
O1—Cu—N1^i^	89.72 (6)	N1—C7—H7	116.9
O1^i^—Cu—N1^i^	90.28 (6)	C2—C7—H7	116.9
N1—Cu—N1^i^	180.0	N2—C8—C9	122.34 (18)
C1—O1—Cu	128.77 (13)	N2—C8—H8	118.8
C7—N1—N2	111.28 (16)	C9—C8—H8	118.8
C7—N1—Cu	123.02 (13)	C14—C9—C10	116.3 (2)
N2—N1—Cu	124.88 (12)	C14—C9—C8	119.63 (19)
C8—N2—N1	113.85 (16)	C10—C9—C8	124.0 (2)
O1—C1—C6	118.78 (19)	C11—C10—C9	121.3 (3)
O1—C1—C2	123.59 (18)	C11—C10—Cl1	117.8 (2)
C6—C1—C2	117.63 (19)	C9—C10—Cl1	120.87 (18)
C1—C2—C3	119.48 (19)	C12—C11—C10	120.1 (3)
C1—C2—C7	122.47 (18)	C12—C11—H11	120.0
C3—C2—C7	117.97 (19)	C10—C11—H11	120.0
C4—C3—C2	121.2 (2)	C11—C12—C13	121.1 (2)
C4—C3—H3	119.4	C11—C12—H12	119.4
C2—C3—H3	119.4	C13—C12—H12	119.4
C3—C4—C5	119.3 (2)	C14—C13—C12	117.9 (3)
C3—C4—H4	120.3	C14—C13—H13	121.1
C5—C4—H4	120.3	C12—C13—H13	121.1
C6—C5—C4	121.4 (2)	C13—C14—C9	123.3 (2)
C6—C5—H5	119.3	C13—C14—Cl2	118.0 (2)
C4—C5—H5	119.3	C9—C14—Cl2	118.66 (16)
C5—C6—C1	121.0 (2)		
			
N1—Cu—O1—C1	25.25 (19)	C1—C2—C7—N1	4.8 (3)
N1^i^—Cu—O1—C1	−154.75 (19)	C3—C2—C7—N1	−178.5 (2)
C7—N1—N2—C8	141.33 (19)	N1—N2—C8—C9	178.03 (18)
Cu—N1—N2—C8	−48.8 (2)	N2—C8—C9—C14	134.7 (2)
Cu—O1—C1—C6	163.39 (17)	N2—C8—C9—C10	−49.5 (3)
Cu—O1—C1—C2	−17.0 (3)	C14—C9—C10—C11	1.1 (3)
O1—C1—C2—C3	179.9 (2)	C8—C9—C10—C11	−174.8 (2)
C6—C1—C2—C3	−0.5 (3)	C14—C9—C10—Cl1	179.69 (17)
O1—C1—C2—C7	−3.5 (3)	C8—C9—C10—Cl1	3.8 (3)
C6—C1—C2—C7	176.1 (2)	C9—C10—C11—C12	−0.4 (4)
C1—C2—C3—C4	−0.9 (3)	Cl1—C10—C11—C12	−179.1 (2)
C7—C2—C3—C4	−177.7 (2)	C10—C11—C12—C13	−0.8 (4)
C2—C3—C4—C5	1.4 (4)	C11—C12—C13—C14	1.2 (4)
C3—C4—C5—C6	−0.6 (4)	C12—C13—C14—C9	−0.5 (4)
C4—C5—C6—C1	−0.9 (4)	C12—C13—C14—Cl2	−178.6 (2)
O1—C1—C6—C5	−179.0 (2)	C10—C9—C14—C13	−0.6 (3)
C2—C1—C6—C5	1.4 (4)	C8—C9—C14—C13	175.5 (2)
N2—N1—C7—C2	−177.83 (19)	C10—C9—C14—Cl2	177.47 (16)
Cu—N1—C7—C2	12.1 (3)	C8—C9—C14—Cl2	−6.4 (3)

Symmetry code: (i) −*x*+1, −*y*+1, −*z*+1.
